# Characterization of Influenza-Like Illness Burden Using Commercial Wearable Sensor Data and Patient-Reported Outcomes: Mixed Methods Cohort Study

**DOI:** 10.2196/41050

**Published:** 2023-03-23

**Authors:** Victoria Hunter, Allison Shapiro, Devika Chawla, Faye Drawnel, Ernesto Ramirez, Elizabeth Phillips, Sara Tadesse-Bell, Luca Foschini, Vincent Ukachukwu

**Affiliations:** 1 Genentech, Inc South San Francisco, CA United States; 2 Evidation Health, Inc San Mateo, CA United States; 3 F. Hoffmann-La Roche Ltd Basel Switzerland; 4 Roche Products Limited Welwyn Garden City United Kingdom

**Keywords:** influenza, influenza-like illness, wearable sensor, person-generated health care data

## Abstract

**Background:**

The burden of influenza-like illness (ILI) is typically estimated via hospitalizations and deaths. However, ILI-associated morbidity that does not require hospitalization remains poorly characterized.

**Objective:**

The main objective of this study was to characterize ILI burden using commercial wearable sensor data and investigate the extent to which these data correlate with self-reported illness severity and duration. Furthermore, we aimed to determine whether ILI-associated changes in wearable sensor data differed between care-seeking and non–care-seeking populations as well as between those with confirmed influenza infection and those with ILI symptoms only.

**Methods:**

This study comprised participants enrolled in either the FluStudy2020 or the Home Testing of Respiratory Illness (HTRI) study; both studies were similar in design and conducted between December 2019 and October 2020 in the United States. The participants self-reported ILI-related symptoms and health care–seeking behaviors via daily, biweekly, and monthly surveys. Wearable sensor data were recorded for 120 and 150 days for FluStudy2020 and HTRI, respectively. The following features were assessed: total daily steps, active time (time spent with >50 steps per minute), sleep duration, sleep efficiency, and resting heart rate. ILI-related changes in wearable sensor data were compared between the participants who sought health care and those who did not and between the participants who tested positive for influenza and those with symptoms only. Correlative analyses were performed between wearable sensor data and patient-reported outcomes.

**Results:**

After combining the FluStudy2020 and HTRI data sets, the final ILI population comprised 2435 participants. Compared with healthy days (baseline), the participants with ILI exhibited significantly reduced total daily steps, active time, and sleep efficiency as well as increased sleep duration and resting heart rate. Deviations from baseline typically began before symptom onset and were greater in the participants who sought health care than in those who did not and greater in the participants who tested positive for influenza than in those with symptoms only. During an ILI event, changes in wearable sensor data consistently varied with those in patient-reported outcomes.

**Conclusions:**

Our results underscore the potential of wearable sensors to discriminate not only between individuals with and without influenza infections but also between care-seeking and non–care-seeking populations, which may have future application in health care resource planning.

**Trial Registration:**

Clinicaltrials.gov NCT04245800; https://clinicaltrials.gov/ct2/show/NCT04245800

## Introduction

### Background

In the United States, an estimated 9 to 41 million annual illnesses are attributable to influenza virus infection, resulting in up to 710,000 hospitalizations and 52,000 deaths annually [1]. Less severe influenza and influenza-like illness (ILI) that do not require hospitalization also cause significant morbidity, the measurement of which historically relied on dedicated studies using self-reported outcomes, often of varying quality [2-5]. Digital syndromic surveillance tools enable the self-reporting of ILI symptoms for influenza and SARS-CoV-2 surveillance on a larger scale, regardless of whether patients sought health care for their ILI [6-9]. Although these tools enable a wider surveillance of ILI outside of traditional health care records, person-generated health data afford means for a more thorough sampling of ILI, unlocking previously inaccessible evidence from non–care-seeking individuals using novel metrics. Commercial wearable sensors passively record health-related data such as step count, exercise intensity, resting heart rate (RHR), and sleep duration and stages. As such, they provide person-generated health data that can be investigated in relation to ILI burden, independently of or in combination with traditional sources of medical data. These data may enhance our understanding of disease states and inform public health and clinical decision-making [10,11].

Using commercial wearable sensors (Fitbit [Fitbit LLC]), we previously demonstrated that nationwide mobility (measured as total daily steps in a US population) decreased owing to ILI symptoms and that ILI burden (determined by the difference in total daily steps) was associated with care-seeking behaviors, the number of workdays missed, and self-reported overall health [12]. Another study showed that abnormalities in RHR and sleep duration, measured using wearable sensors, could be leveraged to predict the real-time incidence of ILI [13]. Recently, wearable sensor data have also been used to assess the physiological signs associated with COVID-19 [14-23].

### Overview of This Study

Here, we investigated the extent to which commercial wearable sensor data (total steps, the proportion of the day spent active, sleep duration, sleep efficiency, and RHR) can be used to assess the severity and clinical course of ILI events. We further characterized the differences in commercial wearable sensor data between individuals who sought health care during their ILI event and those who did not and between those who tested positive for influenza virus infection and those who did not.

## Methods

### Study Design and Participants

#### Overview

**Figure 1:**
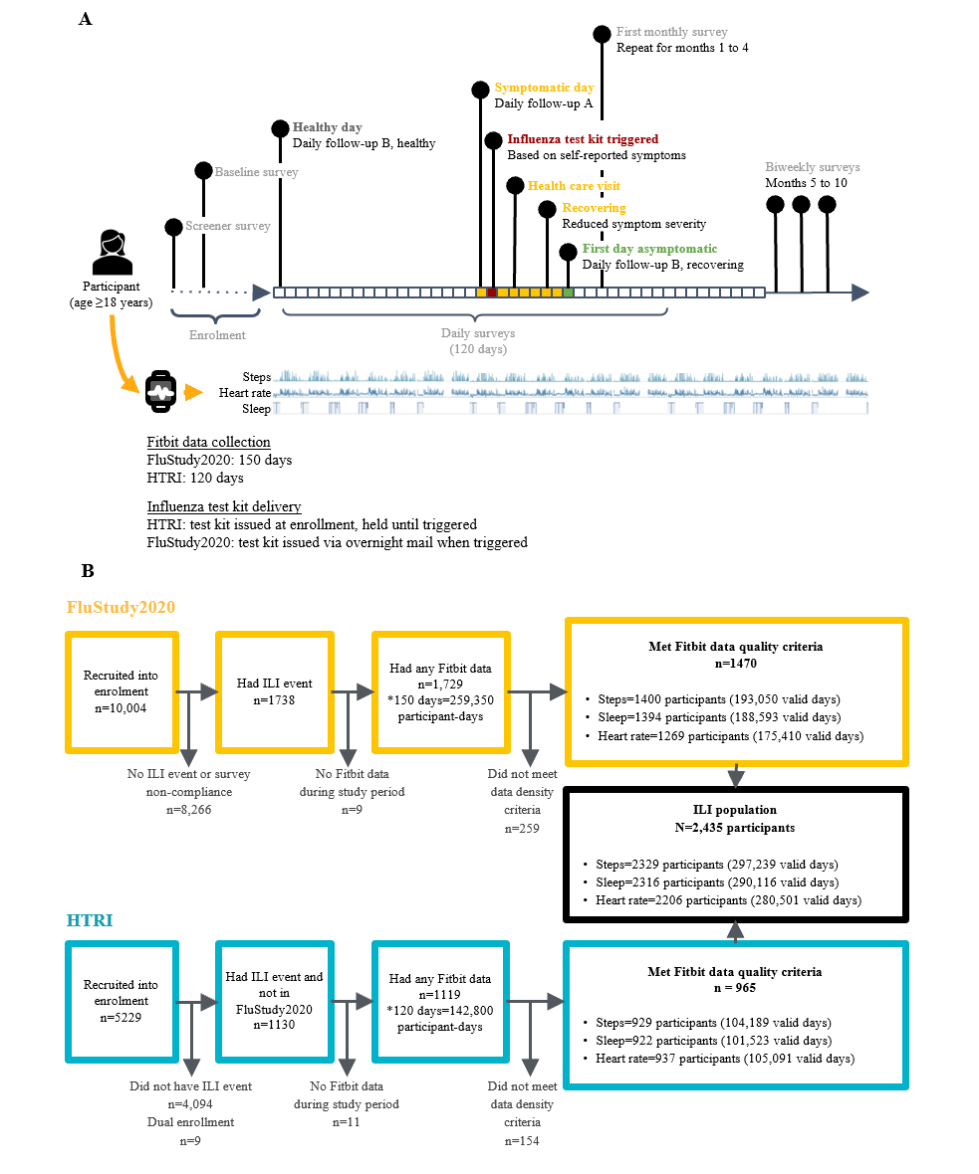
Study design overview and flow diagram for preparing analysis data sets. (A) Study design overview for the FluStudy2020 and Home Testing of Respiratory Illness (HTRI) studies. During the enrollment period, the participants completed a screener survey and a baseline survey and connected their commercial wearable sensor (Fitbit) to the Evidation platform. The participants in the HTRI study were issued with an influenza diagnostic kit during enrollment to be used as instructed based on symptoms reported in the daily survey. Once enrolled, participants completed daily surveys for 120 days, in addition to monthly and biweekly surveys. Wearable sensor data were collected for 150 and 120 days in the FluStudy2020 and HTRI studies, respectively. The participants completed the *Daily follow-up A* survey on days when they reported feeling symptomatic (yellow and red boxes). They completed the *Daily follow-up B*, healthy survey on days when they reported feeling healthy (white boxes), and *Daily follow-up B*, recovering survey on days when they reported feeling healthy but were recovering from a recent influenza-like illness (ILI; green boxes). The labels highlight selected examples of events that the participants could self-report on surveys, as well as study-related activities (such as an influenza diagnostic kit being triggered by the participant’s self-reported symptoms). (B) Participant flow diagram for the FluStudy2020 and HTRI studies.

For a participant to be included in the ILI analysis population, they must have either tested positive for influenza as part of the testing regime in the FluStudy2020 or Home Testing of Respiratory Illness (HTRI) study, met the FluStudy2020 study–defined ILI symptom criteria, or received an antiviral prescription from a health care professional for their ILI event. The FluStudy2020 and HTRI studies were designed independently by different sponsors, but both were performed concurrently by Evidation Health. Both studies were conducted in the United States between December 2019 and October 2020. Given the similarity in study designs (Figure 1A), the data sets were merged and jointly analyzed here to increase the sample size; a participant flow diagram is shown in Figure 1B.

#### FluStudy2020 Design

Participants were enrolled in the FluStudy2020 study if they were aged ≥18 years, lived in the United States, and wore a Fitbit device with heart rate (HR)-tracking capabilities. Participants were excluded from the study if they had been diagnosed with influenza by a health care professional in the previous 3 months.

Before enrollment, the participants completed a baseline survey, which collected data regarding key demographics, medical history, comorbidities, and influenza history (specifically, prior influenza diagnosis, previous influenza treatment, and current influenza vaccination status).

The participants completed daily surveys for the first 120 days and fortnightly surveys for the remainder of the study period. If the participants reported ILI symptoms within the previous 24 hours, detailed symptom, quality of life (QoL), and health care use data were collected through follow-up surveys. To verify the diagnoses, an influenza diagnostic kit (self-administered nasal swab [Molecular Testing Labs]) was sent to the participants who experienced fever, at least 1 respiratory symptom (cough, sore throat, or nasal congestion), and at least 1 systemic symptom (headache, muscle or joint ache, chills, or fatigue). The diagnostic criteria were based on those highlighted by precedents in the literature and influenza clinical trials [24-26]. Data on total steps, active time, HR, and sleep were collected through continuous passive monitoring using a Fitbit.

#### HTRI Study Design

Participants were enrolled in the HTRI study prospectively and responded to daily surveys about their symptoms, medical experiences, etc. Many of the questions used a similar or identical language to that used in the surveys conducted in FluStudy2020. However, there were some notable differences between the 2 study designs (Table 1).

**Table 1:** FluStudy2020 and Home Testing of Respiratory Illness (HTRI) study designs.

	FluStudy2020	HTRI study
Influenza test kit	Sent to the participants immediately after they reported relevant symptoms in a daily survey The participants could complete multiple test kits	Sent to the participants during enrollment; they completed the test only when prompted after they reported relevant symptoms in a daily survey The participants could only submit a sample upon their first trigger
Influenza test kit trigger	Fever At least 1 respiratory symptom (cough, sore throat, or nasal congestion) At least 1 systemic symptom (headache, muscle or joint ache, chills, or fatigue)	Cough At least one of the following symptoms: fever, sweats, chills or shivering, body or muscle aches
Health care visits	The participants were asked whether they made a health care visit in their daily surveys	The participants were asked whether they made a health care visit in a recovery survey, which was completed 14 days after the influenza test kit criteria were met and captured the dates of ILI^a^ onset and recovery

^a^ILI: influenza-like illness.

Given these differences in study design, the current analysis excluded participants who tested positive for influenza in the HTRI study but did not meet any of the FluStudy2020 ILI population criteria. There was also a small subset of HTRI participants whose symptoms would have met the influenza test kit criteria for FluStudy2020 but from whom a sample was not collected because they did not meet the HTRI influenza test kit criteria. To combine the HTRI data set with the FluStudy2020 data set, it was necessary to verify that the HTRI participants’ self-reported illness dates (which were provided in the same recovery survey in which a health care visit was reported) aligned with the analysis-derived ILI event dates (which were identified during the analysis of the daily survey responses). This permitted the verification that only health care visits made during the same illness period as the ILI event period were included in the analysis. The HTRI participants were categorized as having made or not made a health care visit only if their self-reported ILI event period overlapped with the analysis-derived ILI event period.

### Analysis of ILI-Related Changes in Fitbit Data

Before the analysis, wearable sensor data were assessed for quality and completeness in 3 steps. First, minute-level streams were assessed for their physiological plausibility. Second, participant days (day-level aggregates of minute-level streams) were assessed for wear time and data availability, excluding missing minutes with null or physiologically implausible values from wear-time estimates. Third, the participants’ Fitbit data density was assessed, excluding days with insufficient wear time or unavailable data from consideration. Only the participants who met the data density criteria were eligible for inclusion in the analyses of the relevant data stream.

In the first step, physiologically implausible values were removed. Minutes with >400 steps were removed from the steps stream. Minutes with <30 beats per minute (bpm) or >220 bpm were removed from the HR stream. In the second step, HR data were considered valid on days with available Fitbit-estimated daily RHR and at least 10 hours of HR wear time (the sum of the minutes with nonzero, nonmissing HR values), consistent with the standard wear-time criteria [27]. Steps data were considered valid on days with valid HR wear time or at least 10 hours of steps wear time, which was assessed on a rolling basis; steps nonwear periods were 180 consecutive minutes with missing values or 0 steps value. This definition was consistent with standard criteria [25], except for the modification of the nonwear threshold from 60 to 180 minutes to accommodate our population, who may have more prolonged periods of 0 steps during ILI events. Sleep data were considered valid on days with at least 1 minute of total sleep time. In the third step, each participant’s day-level Fitbit time series was indexed on the date of ILI onset, which is hereon denoted as day 0. Then, the participants’ Fitbit data density was assessed during 4 time periods of interest to ensure that only those with sufficient coverage were included in the analyses. Valid data were required on >50% of days during the ILI event and ≥10% of each day of the week during the baseline period, following previously published methods [20]. The 50% of days during the ILI were broken down as follows: to ensure that the participants were observed during the latent period of the ILI event, at least 1 valid day was required from days −1 to −4 relative to ILI onset; to ensure that the participants were observed at ILI onset, valid data were required on day 0; and to ensure that dense data were available throughout the ILI event, particularly during the earliest days when symptoms were expected to be most severe, it was required that the participants had ≥6 valid days during the symptomatic period (days +1 to +9), including at least 1 valid day from days +1 to +3. The baseline period criteria (ie, valid data on 10% of all baseline Mondays, 10% of all baseline Tuesdays, etc) were enforced to accommodate the baseline model, which included a term for the day of the week. The above criteria were applied on a per-channel basis, making it possible for a participant to be included in the analysis of steps data but not RHR, for example.

Before enforcing the data quality criteria, there were 2848 potential participants with at least 1 day of Fitbit data. In total, 14.5% (413/2848) of participants did not meet the data density criteria for any of the 3 channels; the remaining 85.5% (2435/2848) of participants comprised the final ILI analysis population. Overall, 2329 (95.6%) participants had 297,239 participant-days with valid Fitbit steps data (92.5% of 321,480 possible days [{1400 FluStudy2020 participants with valid steps data × 150 study days} + {929 HTRI participants × 120 study days}]); 2316 (95.1%) participants had 290,116 participant-days with valid Fitbit sleep data (90.7% of 319,740 possible days); and 2206 (90.6%) participants had 280,501 participant-days with valid Fitbit HR data (92.2% of 302,790 possible days).

Five day-level aggregate features hypothesized to be relevant to ILI were computed from the minute-level Fitbit data: total daily steps (ie, the total number of steps taken in a day), the proportion of the day that the participant spent being physically active (≥50 steps per minute), daily RHR (ie, Fitbit-estimated HR while at rest), total sleep duration (ie, Fitbit-estimated total length of time the participant spent sleeping), and sleep efficiency (ie, Fitbit-estimated proportion of the main sleep interval spent asleep [minutes asleep / total time spent in bed − minutes spent in bed after waking up], which range from 0 to 100). In the time to return to baseline (TTRB) analysis, day-to-day changes in RHR (RHR δ) were also included to reduce the impact of autocorrelation of RHR measurements. The day-level features were winsorized, clipping extreme values to the 99.95th percentile value (taken across all participant-days).

### Baseline Model

A model comparison procedure was performed to identify relevant terms for the baseline model using the dredge function of the *MuMIn* package in R (R Foundation for Statistical Computing) [28]. Subsequently, the following linear mixed-effects regression model was fit to day-level Fitbit features from healthy days (ie, excluding days −4 to +9 relative to the participant’s ILI onset):

[Wearable sensor measure] ≈ β0 + β1 × week + β2 × week2 + β3 × week3 + β4 × day of week + β5 × regional shelter in place (SIP) + u0 + ε

The data input into the model comprised a panel with 1 row per participant day (ie, n valid participants × m study days, where the number of valid participants was determined by the number of participants meeting density criteria for the relevant channel, and the number of study days was determined by the number of study days with valid wear time for each of the n participants). The model included 3 fixed effects for the first, second, and third expansion of the week of the year (denoted by “week,” “week^2^,” and “week^3^”), where a week refers to one of the first 7 dates on which activity data were available across the analysis population and is incremented until the final date on which activity data were collected across all participants. Categorical fixed effects were specified for the day of the week (7 levels, one for each day of the week; denoted by “day of week”) and for regional SIP orders related to COVID-19 (denoted by “regional SIP”; 4 levels: no order, advisory or recommendation, mandatory for at-risk individuals, and mandatory for all individuals), which were specific to the participant’s geographic region of residence and the calendar date. A random intercept was specified on the participant ID to control for individual differences in average activity levels (denoted by “u”). This model was repeated 5 times, once for each wearable sensor measure. Residuals were computed as the difference between the model-estimated wearable sensor measure value and the observed value for each participant day.

### Day-by-Day and Cumulative ILI Burden

Day-by-day ILI burden was defined as the residual variance in behavior on days −4 to +9 that was not accounted for in the baseline model and was computed separately for each of the 5 Fitbit features examined in this study. Cumulative ILI burden was calculated as the sum of residuals across days −4 to +9. Missing residuals were imputed with an individual-level exponentially weighted rolling mean for the purpose of computing only the cumulative ILI burden. The cumulative ILI burden was computed for the total number of steps and total sleep duration, as a cumulative sum would not have a meaningful interpretation for the other features. Our original analyses included 2 other aggregate metrics for ILI burden: the sum of residuals across days −1 to +1 and the residual on day 0 only. These results were omitted in the interest of reducing redundancy, as they closely mirrored the results of the cumulative burden across days −4 to +9. However, *P* values from the statistics for the cumulative ILI burden across days −4 to +9 were still Bonferroni corrected for all 3 methods to reduce the likelihood of reporting false positives.

### Defining the Baseline Period for TTRB Metrics

When computing the TTRB, the baseline activity period for each participant was defined as any time before the latent ILI phase (ie, at least 5 days before ILI onset) or more than 21 days after ILI onset. To mitigate the potential effects of COVID-19–related SIP orders, ILI events were separated according to whether they occurred before, on, or after March 15, 2020. For the former, the baseline period was additionally restricted to before March 15, 2020, and for the latter, the baseline period was restricted to after March 15, 2020.

### TTRB Activity Levels

This analysis was used to estimate the time at which the participants resumed their typical healthy behavior patterns following an ILI event (ie, the TTRB). The analysis was performed on residuals from the baseline model. Residuals were smoothed with a rolling centered 3-day mean to reduce spurious patterns that could interfere with the TTRB.

The TTRB was computed as follows: for each individual and each activity feature, the mean and SD during the baseline period were calculated. Then, all days within 1× SD of the individual’s baseline mean were considered to be within the healthy range of behavior. Starting with the day of ILI onset, the first of 2 consecutive days on which the activity feature was within the range of healthy behavior (within 1× SD of baseline activity) was determined. For RHR, the TTRB was computed for each individual by taking the mean and SD of RHR. Then, the first day that was followed by 14 days that had <32% of values outside the healthy range (greater than 1× SD from baseline activity) was identified. Our original analyses included 6 different methods of computing the TTRB, and 3 smoothing functions were applied to the residuals. As with the cumulative ILI burden, only 1 TTRB method and 1 smoothing function per activity feature are reported here, and redundant results have been omitted. *P* values were Bonferroni corrected for all 18 TTRB method × smoothing function combinations to reduce the false-positive rate.

### Differences in ILI Burden and TTRB Between Participant Cohorts

The day-by-day deviations from typical healthy behavior, cumulative ILI burden, and TTRB were compared between the participants who did and did not seek health care as well as between those with confirmed influenza infection and those with ILI symptoms only.

Differences between the cohorts in the day-by-day residuals were assessed using linear mixed-effects regression. The dependent measures were day-level residuals from days −4 to +9 and an additional measurement for baseline, which was the mean residual across healthy days. The model included a categorical fixed effect for ILI day (15 levels, one for each day from days −4 to +9 and an intercept for baseline) and a categorical fixed effect for cohort membership (health care seeker vs nonseeker for one model and confirmed influenza vs ILI symptoms only for the other model). A random intercept was specified on the participant ID. This model was repeated 10 times, once for each combination of the 5 activity features and 2 cohort comparisons. A total of 14 pairwise contrasts were computed per model; each compared the change in residuals from baseline between the 2 cohorts (ie, cohort 1 [flu day − baseline] − cohort 2 [flu day − baseline]). *P* values were Bonferroni corrected for 14 tests per cohort comparison.

Differences between the cohorts in the cumulative ILI burden were assessed using Welch 2-sample *t* tests (2-tailed). *P* values were Bonferroni corrected for 3 tests per cohort comparison.

Differences between the cohorts in the TTRB were calculated using the Mann-Whitney *U* test, and *P* values were Bonferroni corrected for each activity feature separately with 36 different tests (3 smoothing conditions × 6 TTRB methods × 2 cohort comparisons).

### Digital Correlates of Patient-Reported Outcomes

Pairwise correlations were run to assess the direction, strength, and significance of the relationship between digital measures of ILI and subjective measures of ILI from patient-reported outcomes (PROs). Spearman rank-order correlations were used to accommodate skewed distributions of digital measures and PROs. *P* values were false discovery rate corrected for 3105 tests following the Benjamini-Hochberg procedure: 23 PRO variables × 135 wearable sensor variables ([126 TTRB features comprising 7 sensor data features originally explored × 3 smoothing functions × 6 TTRB computation methods] + [9 cumulative burden features comprising 3 sensor data features originally explored × 3 cumulative burden windows]). Only a subset of these features is presented here in the interest of parsimony and redundancy reduction.

### Software

R and Python standard packages were used to compile the data sets and prepare them for analysis. Statistical analyses were performed in R using the following packages: *stats* (version 3.6.4), *lme4* (version 1.1-25), *Hmisc* (version 4.5.0), *effects* (version 4.2-0), and *gtsummary* (version 1.3.5). Visualizations were created in R using the package *ggplot2* (version 3.3.0).

### Ethics Approval

The FluStudy2020 protocol was reviewed and approved by the institutional review board of the WIRB-Copernicus Group (study number: 1271500). Written informed consent was obtained from all the participants before their enrollment in the study. The HTRI study (NCT04245800) protocol was approved by the institutional review board of the WIRB-Copernicus Group (study number: 1271380), and written informed consent was obtained from all the participants before their enrollment in the study [29].

## Results

### Demographics and Baseline Characteristics

At recruitment, 10,004 individuals were enrolled in FluStudy2020, of whom 1738 (17.4%) experienced an ILI event during the study period. Of these 1738 participants, 1470 (84.6%) had wearable sensor data from at least 1 channel (steps, sleep, or HR) that were valid for inclusion in the analysis (refer to *Methods* for data quality criteria) and were included in the final ILI population (ie, the analysis population). A total of 5229 participants were enrolled in the HTRI study, of which 8 (0.2%) were concurrently enrolled in FluStudy2020; therefore, only their data from FluStudy2020 were considered for analysis. Of the remaining 99.8% (5221/5229) HTRI participants, 21.6% (1130/5221) experienced an ILI event, among whom 85.4% (965/1130) had wearable sensor data from at least 1 channel that were valid for inclusion in the analysis.

After combining the FluStudy2020 and HTRI data sets, the final ILI population comprised 2435 participants (number of patients with analyzable data per channel: steps, n=2329, 95.6%; sleep, n=2316, 95.1%; and HR, n=2206, 90.6%). Most participants in the final ILI population were aged 18 to 49 years (2183/2435, 89.7%), female (2080/2435, 85.4%), and White (2175/2435, 89.3%; Table 2).

The ILI population included 14.9% (364/2435) of participants who tested positive for influenza with an at-home test provided by the study and 83.1% (2024/2435) of participants whose self-reported symptoms met the prespecified clinical diagnostic criteria of ILI: experiencing a combination of fever, at least 1 respiratory symptom, and at least 1 systemic symptom. The remaining 1.9% (47/2435) of participants were included in the ILI population because they self-reported having been diagnosed with influenza by their physician or self-reported having been prescribed an antiviral medication. Among the participants who reported care-seeking behavior during their illness (1988/2435, 81.6%), 31.1% (618/1988) of participants reported making a health care visit.

**Table 2:** Baseline demographics and clinical characteristics of the study participants.

Characteristics		Overall (n=2435), n (%)	FluStudy2020 (n=1470), n (%)	HTRI^a^ study (n=965), n (%)
**Age (years)**				
	18-49	2183 (89.65)	1312 (89.25)	871 (90.26)
	50-64	236 (9.69)	148 (10.07)	88 (9.12)
	>65	16 (0.66)	10 (0.68)	6 (0.62)
**Sex**				
	Male	342 (14.05)	112 (7.62)	230 (23.83)
	Female	2080 (85.42)	1347 (91.63)	733 (75.96)
	Nonbinary	13 (0.53)	11 (0.75)	2 (0.21)
**Race**				
	American Indian or Alaska Native	7 (0.29)	3 (0.2)	4 (0.41)
	Asian	64 (2.63)	27 (1.84)	37 (3.83)
	Black or African American	77 (3.16)	53 (3.61)	24 (2.49)
	Native Hawaiian or Other Pacific Islander	3 (0.12)	3 (0.2)	0 (0)
	White	2175 (89.32)	1311 (89.18)	864 (89.53)
	Multiple races	84 (3.45)	55 (3.74)	29 (3.01)
	Other	25 (1.03)	18 (1.22)	7 (0.73)
**American region**				
	Northeast	441 (18.11)	258 (17.55)	183 (18.96)
	South	727 (29.86)	465 (31.63)	262 (27.15)
	Midwest	800 (32.85)	485 (32.99)	315 (32.64)
	West	466 (19.14)	261 (17.76)	205 (21.24)
	Unknown	1 (0.04)	1 (0.07)	0 (0)
**BMI (kg/m^2^)**				
	Underweight (<18.5)	22 (0.9)	17 (1.16)	5 (0.52)
	Normal (18.5-24.9)	553 (22.71)	326 (22.18)	227 (23.52)
	Overweight (25.0-29.9)	679 (27.89)	395 (26.87)	284 (29.43)
	Obese (≥30.0)	1179 (48.42)	730 (49.66)	449 (46.53)
	Unknown	2 (0.08)	2 (0.14)	0 (0)
**Health care visit**				
	Yes	618 (25.38)	430 (29.25)	188 (19.48)
	No	1380 (56.67)	1001 (68.10)	379 (39.27)
	Unknown	437 (17.95)	39 (2.65)	398 (41.24)
**Influenza status^b^**				
	Positive	364 (14.95)	193 (13.13)	171 (17.72)
	Symptom criteria only	2024 (83.12)	1238 (84.22)	786 (81.45)
	Unknown	47 (1.93)	39 (2.65)	8 (0.83)
**Prescribed antiviral**		276 (11.33)	182 (12.38)	94 (9.74)
	Baloxavir marboxil	31 (1.27)	21 (1.43)	10 (1.04)
	Oseltamivir phosphate	249 (10.23)	164 (11.16)	85 (8.81)
**Data validity**				
	Valid steps data	2329 (95.65)	1400 (95.24)	929 (96.27)
	Valid heart rate data	2206 (90.6)	1269 (86.33)	937 (97.1)
	Valid sleep data	2316 (95.11)	1394 (94.83)	922 (95.54)

^a^HTRI: Home Testing of Respiratory Illness.

^b^Defined using symptom criteria.

### Characterization of ILI Burden Using Wearable Sensor Measures: Steps

Across the study period, the ILI population took an average of 8019 daily steps and spent 15% of the nonresting portion of their day active with >50 steps per minute (out of all the minutes with nonzero steps), which is hereafter referred to as active time.

**Figure 2:**
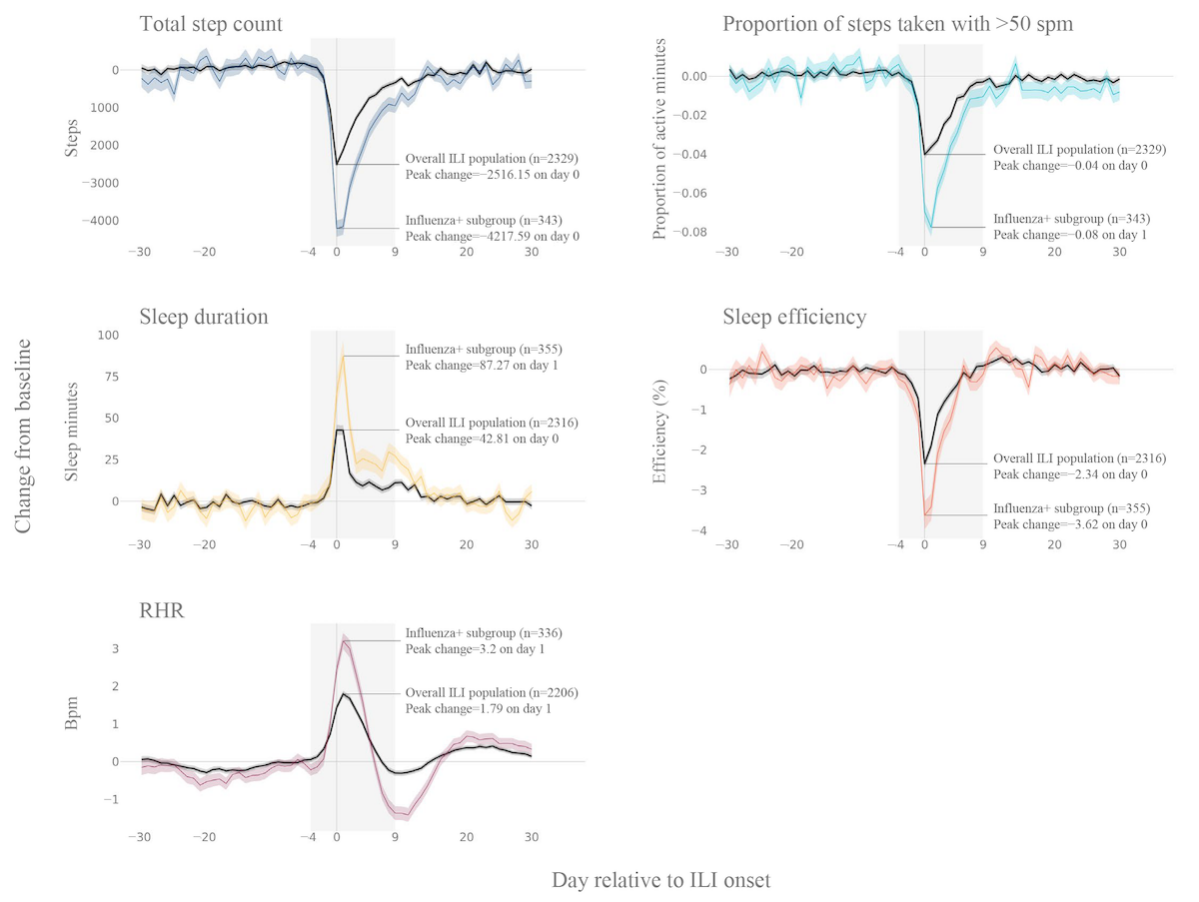
Deviations from baseline levels across wearable measures during an influenza-like illness (ILI) event. Day-by-day group mean (SE) changes from baseline values for 5 wearable sensor features derived from day-level sensor data are shown. “Steps total” was the total number of steps taken per day. “Total steps count” was the total number of steps taken per day. “Proportion of steps taken with >50 spm” was the ratio of minutes in the day that the participant took over 50 steps per minute (spm) relative to the total number of minutes with at least 1 step. “Sleep duration” was the total number of minutes in the day that the participant spent asleep. “Sleep efficiency” was the ratio of minutes the participant was asleep relative to the total minutes spent in bed until the end of the main sleep event (obtained from the Fitbit application programming interface). “RHR” was heart rate (beats per minute [bpm]) during periods of inactivity (obtained from the Fitbit application programming interface). Gray lines illustrate values for the entire wearable analysis population available for that wearable sensor feature; colored lines represent values for the subset of the analysis population who tested positive for influenza. The shaded region covers days −4 to +9 relative to ILI onset, which was the time window used in statistical analyses of day-by-day ILI burden. Influenza+: influenza-positive; RHR: resting heart rate.

Participants saw a change in their total daily steps during ILI events. Just before ILI onset, daily step counts began decreasing from baseline values, with a peak change observed on day 0 (day of symptom onset), when participants lost a mean of 2516 daily steps (Figure 2). This pattern was exacerbated in the subset of participants with a positive influenza test, who lost a mean of 4218 steps on day 0 (Figure 2). In statistical comparisons of ILI days with baseline (entire ILI population included), the difference in total daily steps was significantly negative across days −1 to +9 (*P*<.005; Table S1 in Multimedia Appendix 1). The cumulative ILI burden (ie, the summed daily deviations from baseline) amounted to a mean loss of 12,594 (SD 26,527; median −11,108, IQR −26,633 to 2372; Figure S1 in Multimedia Appendix 1) steps, and the median TTRB for total daily steps following an ILI event was 3 (IQR 1-6) days.

Active time also decreased from baseline values during ILI events. Across the ILI population, the peak mean change in active time was −4% of the total daily minutes, which was observed on days 0 and 1 (Figure 2). In the subset of participants with a positive influenza test, the peak mean change in active time was −8% on day 1 (Figure 2). In statistical comparisons, active time was significantly less than the baseline values from days −1 to +6 relative to ILI onset (*P*<.001; Multimedia Appendix 1, Table S1). The median TTRB for active time following an ILI event was 3 (IQR 1-6) days.

### Characterization of ILI Burden Using Wearable Sensor Measures: Sleep

Across the study period, participants in the ILI population slept an average of 427 minutes (7.1 hours) per day, with an average sleep efficiency score of 89.2%.

Sleep duration changed during ILI events. Shortly before ILI onset, sleep duration began to increase from baseline values, with the peak change occurring on day 0 when participants slept 43 minutes more than baseline values on average across the ILI population. In the participants who tested positive for influenza, the peak change occurred on day +1 when they slept 87 minutes more than baseline values (Figure 2). Statistical test results revealed a significant increase in sleep duration across the ILI population on days −1 to +5 and on day +9 compared with baseline values, with a peak statistical increase by 43 minutes occurring on days 0 and +1 (95% CI 37-49; *P*<.001; Table S1 in Multimedia Appendix 1). The cumulative ILI burden in sleep time amounted to a mean increase of 182.7 (SD 542.4) minutes across the ILI event (median 150.1, IQR −124 to 476; Figure S1 in Multimedia Appendix 1), and the median TTRB for daily sleep duration following an ILI event was 3 (IQR 1-5) days.

Sleep efficiency decreased from baseline levels during ILI events, beginning on day −1 and peaking on day 0, when sleep efficiency was 2.34% lower than baseline levels on average across the ILI population. In the participants who tested positive for influenza, the peak change was −3.62%, which also occurred on day 0 (Figure 2). In statistical tests, the negative change in sleep efficiency observed in the ILI population was significant across days −1 to +4 (*P*<.001), with a peak statistical difference of −2.3 on day 0 (*P*<.001; Table S1 in Multimedia Appendix 1). The median TTRB for sleep efficiency following an ILI event was 3 (IQR 1-5) days.

### Characterization of ILI Burden Using Wearable Sensor Measures: RHR

The mean RHR across the study period was 68 bpm. RHR began to rise above baseline levels shortly before ILI onset and remained elevated for several days during the ILI event (Figure 2). RHR fell below baseline levels on day +8 before a secondary, smaller increase to above baseline levels, which lasted longer than the initial elevated phase. The RHR of participants was elevated significantly above baseline levels on days −2 to +6 (*P*≤.001) and fell significantly below baseline levels on days +8 and +9 (*P*=.002 and *P*<.001, respectively; Table S1 in Multimedia Appendix 1). The greatest change from baseline levels was 1.8 bpm, which was observed on day +1 (Figure 2). In the subset of participants who tested positive for influenza, the peak increase in RHR was 3.2 bpm (Figure 2). Across the entire ILI population, the median TTRB for RHR was 10 (IQR 1-22) days, and the median TTRB for RHR δ (the change in RHR from the preceding day) was 6 (IQR 1-15) days.

### Digital Correlates of PROs

**Figure 3:**
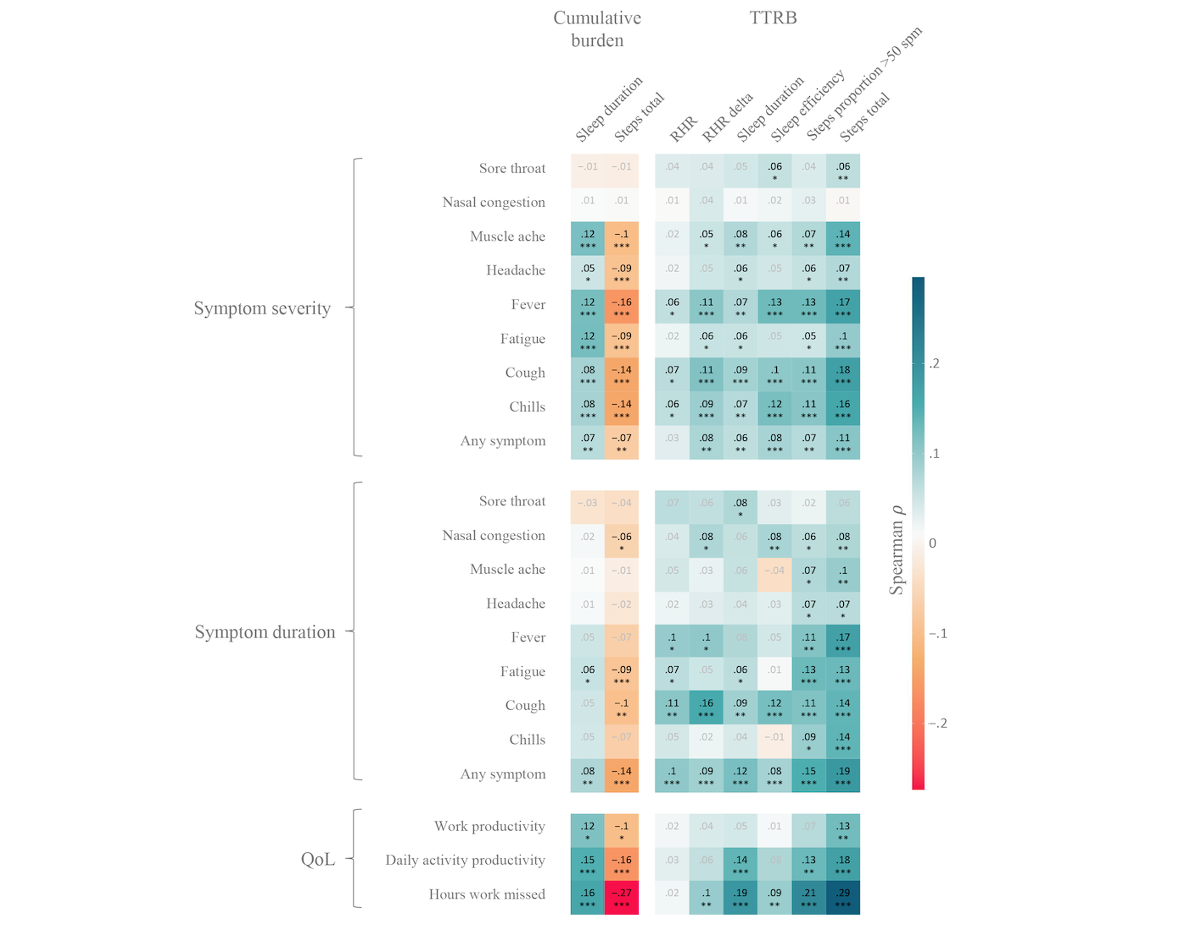
Correlations between patient-reported outcomes (y-axis) and wearable sensor data (x-axis). The color shading and the value printed in each cell indicate Spearman ρ correlation coefficient for the association between the variables in the corresponding row and column. *P* values of statistical associations were false discovery rate corrected for 3105 total tests (not all depicted here), following the Benjamini-Hochberg procedure. Associations that reached statistical significance are illustrated with more intense color and are annotated with an asterisk (*P<.05; **P<.01; ***P<.001). QoL: quality of life; RHR: resting heart rate; RHR δ: the change in RHR from the previous day; spm: steps per minute; TTRB: time to return to baseline.

Wearable sensor data showed a statistically significant but weak correlation with patient-reported severity and duration of key ILI symptoms, as well as QoL outcomes. The total ILI burden on daily step count and sleep duration over the course of an ILI event correlated with patient-reported severity of fever, cough, headache, muscle ache, chills, fatigue, and QoL outcomes (hours of missed work; Figure 3). Wearable measures of illness duration were derived based on the TTRB for each wearable sensor feature, which characterized the duration of ILI-related deviations from the participants’ typical day-to-day activity patterns. TTRB measures also showed a statistically significant but weak correlation with patient-reported symptom duration and symptom severity for key ILI symptoms, including fever; cough; muscle aches; chills; fatigue; and QoL measures, notably hours of missed work (Figure 3).

### Cohort Comparison: Wearable Measures of ILI Burden and TTRB in Participants Who Sought Health Care Compared With Those Who Did Not

The participants who sought health care were defined as those who self-reported that they received primary or urgent care for their ILI. Demographics and baseline characteristics were similar between the participants who sought health care for their ILI event and those who did not, except that those who sought health care tended to be older (85/618, 13.8% were aged ≥50 years) than those who did not (126/1380, 9.1% were aged ≥50 years; Table S2 in Multimedia Appendix 1). The proportion of participants with a positive influenza test result during the study was greater among those who sought health care than among those who did not (160/618, 25.9% vs 197/1380, 14.3%).

**Figure 4:**
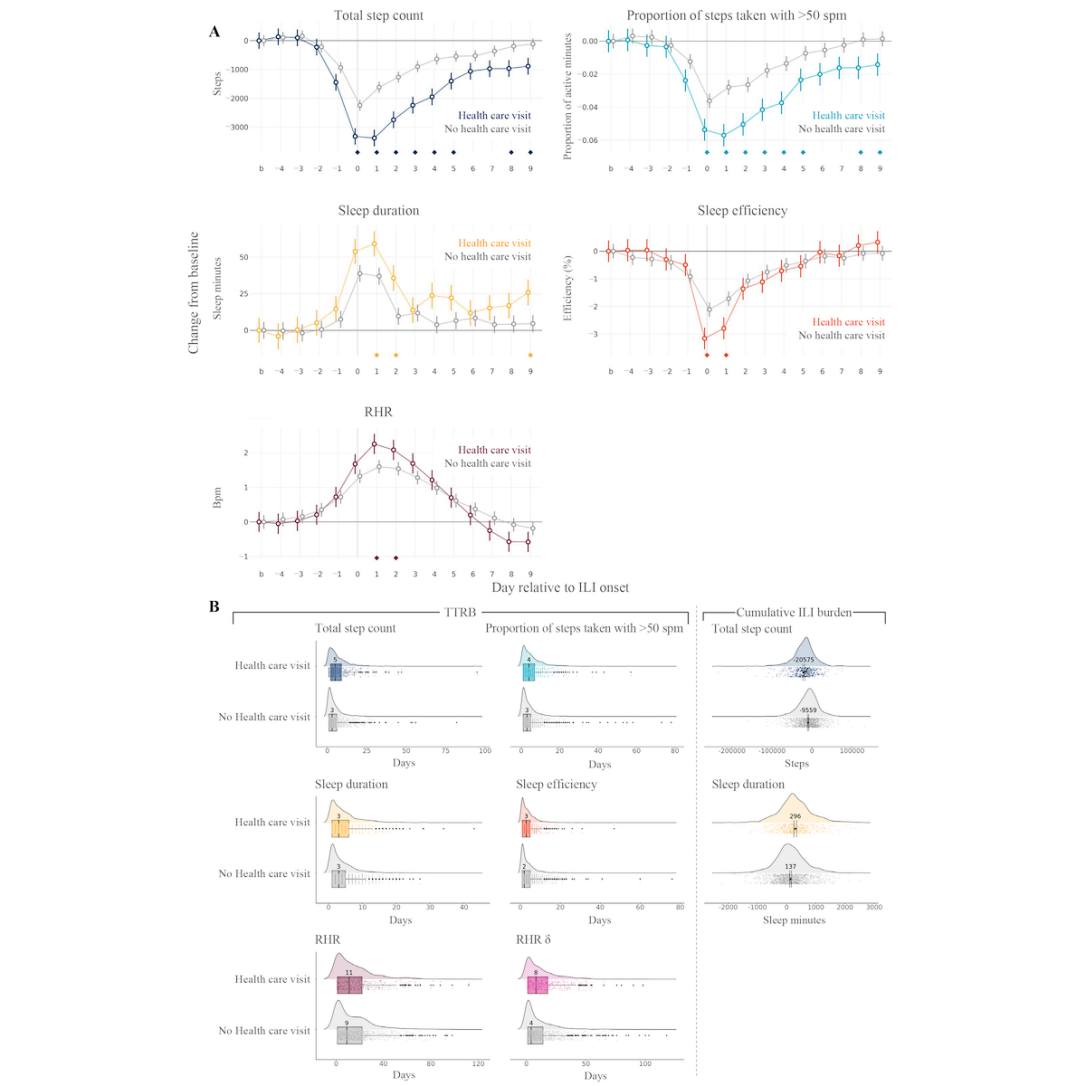
Daily influenza-like illness (ILI) burden and time to return to baseline (TTRB) in health care–seeking versus non–health care–seeking populations. Colored data (plot points, lines, and boxes) represent health care–seeking participants; gray lines represent non–health care–seeking participants. (A) Results of regression analyses of day-by-day ILI burdens of health care–seeking and non–health care–seeking cohorts are shown; separate regression analyses were conducted for each wearable sensor feature. Plotted points are model-fitted estimates (95% CI error bars) for each cohort, on each day of the ILI window and for the baseline period (computed as the mean across all healthy days; represented by b on the x-axis). Diamonds above the x-axis indicate contrasts reaching statistical significance after Bonferroni correction for 14 tests; contrasts were the difference in ILI burden between day n and baseline for the health care–seeking cohort compared with the non–health care–seeking cohort. (B) TTRB and cumulative ILI burden for each sensor feature are shown for individual participants (cloud plots) and overall (density distributions) for health care–seeking and non–health care–seeking cohorts. For TTRB (first 2 columns), box plots (center line, median; box limits, upper and lower quartiles; points, outliers) are overlaid and annotated with the median value for the corresponding population. For cumulative ILI burden (third column), mean and 95% CI (point with error bars) are overlaid and annotated with the mean value. bpm: beats per minute; RHR: resting heart rate; RHR δ: the change in RHR from the previous day; spm: steps per minute.

Day-by-day ILI burden for participants who sought health care versus those who did not are shown for each wearable sensor feature in Figure 4A. The peak loss of daily steps was 1765 steps greater in the participants who sought health care than in those who did not (Table S3 in Multimedia Appendix 1). Furthermore, care seekers lost significantly more active time than non–care seekers (Table S3 in Multimedia Appendix 1). A significant difference in the mean overall ILI burden on total daily steps was observed between the participants who sought health care and those who did not (Δ −11,016; 95% CI −13,676 to −8356; *P*<.001; Figure 4B). Health care seekers also experienced a significantly greater ILI burden on sleep duration on days +1, +2, and +9 than those who did not seek health care (Figure 4A). The change in mean sleep duration from baseline over the course of the ILI event was 159 minutes more for health care seekers than for participants who did not seek health care (Δ 159, 95% CI 103.7-214.1; *P*<.001; Figure 4B). Sleep efficiency scores were significantly lower for those who sought health care than for those who did not on days 0 and +1 (Figure 4A). In addition, those who sought care experienced significantly greater RHR elevations from baseline on days 0 and +1 than those who did not seek care (Figure 4A).

Finally, the participants who sought health care tended to have a longer TTRB for wearable sensor features after ILI onset than those who did not seek health care (Figure 4B); TTRB was 2 and 4 days longer for total daily steps (*U*=291,474.5; *P*≤.001) and the day-to-day change in RHR (RHR δ; *U*=294,092; *P*<.001), respectively, for health care seekers.

### Cohort Comparison: Wearable Measures of ILI Burden and TTRB in Participants With Confirmed Influenza Infection Compared With Those Reporting ILI Symptoms Only

**Figure 5:**
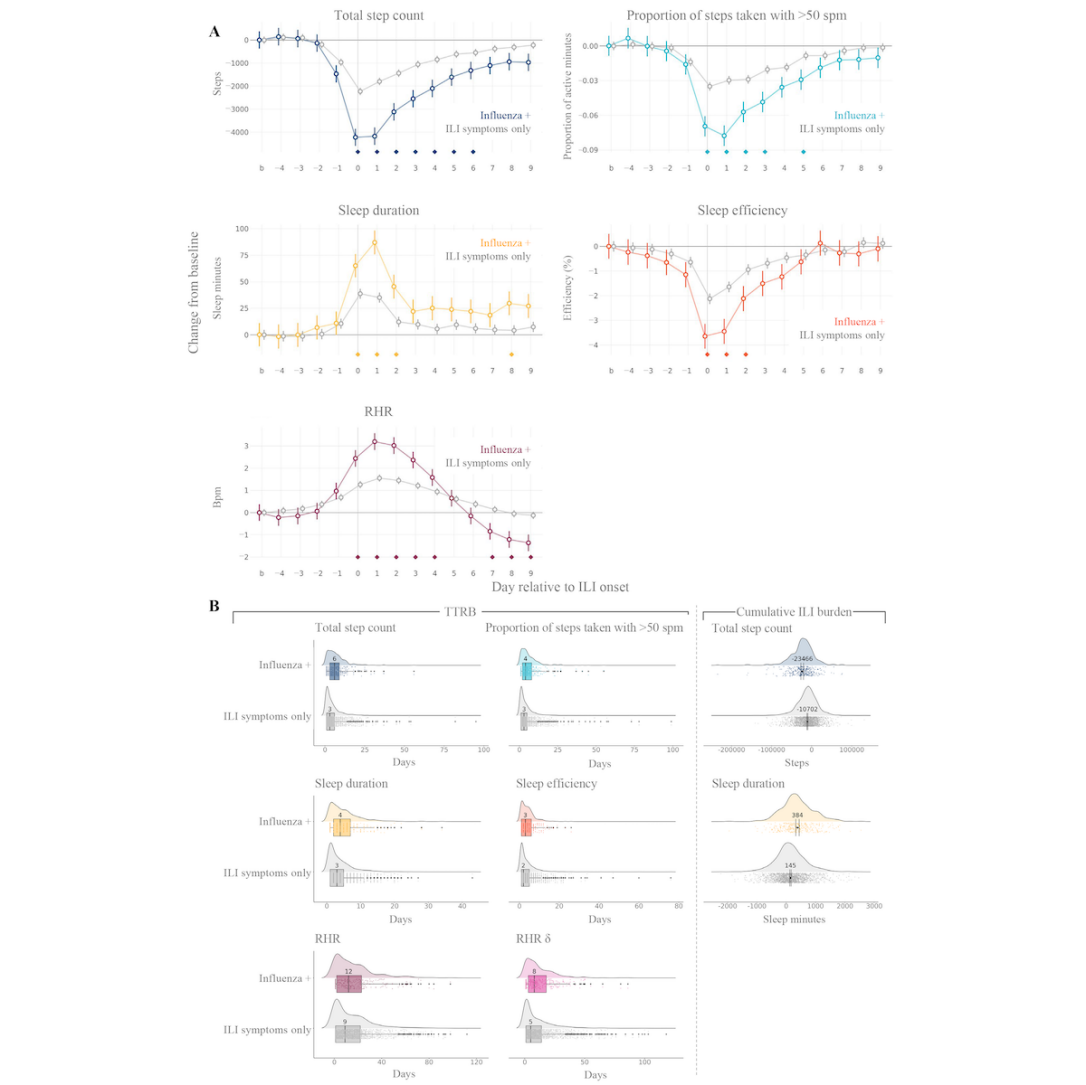
Daily influenza-like illness (ILI) burden and time to return to baseline (TTRB) in the participants with confirmed influenza infection versus those with ILI symptoms only. Colored data (plot points, lines, and boxes) represent the participants who were influenza positive (influenza+); gray lines indicate those with ILI symptoms only. (A) Results of regression analyses of day-by-day ILI burdens of influenza+ and ILI symptoms–only cohorts are shown; separate regression analyses were conducted for each wearable sensor feature. Plotted points are model-fitted estimates (95% CI error bars) for each cohort, on each day of the ILI window and for the baseline period (computed as the mean across all healthy days; represented by b on the x-axis). Diamonds above the x-axis indicate contrasts reaching statistical significance after Bonferroni correction for 14 tests; contrasts were the difference in ILI burden between day n and baseline for the influenza+ cohort compared with the ILI symptoms–only cohort. (B) TTRB and cumulative ILI burden for each sensor feature are shown for individual participants (cloud plots) and overall (density distributions) for influenza+ and ILI symptoms–only cohorts. For TTRB (first 2 columns), box plots (center line, median; box limits, upper and lower quartiles; points, outliers) are overlaid and annotated with the median value for the corresponding population. For cumulative ILI burden (third column), the mean and 95% CI (point with error bars) are overlaid and annotated with the mean value. bpm: beats per minute; influenza+: influenza-positive; RHR: resting heart rate; RHR δ: the change in RHR from the previous day; spm: steps per minute.

Demographics and baseline characteristics were similar between participants with confirmed influenza infection during the study (influenza-positive cohort) and those with ILI symptoms only (symptoms-only cohort; Table S4 in Multimedia Appendix 1). Day-by-day ILI burden for the influenza-positive and symptoms-only cohorts is shown for each wearable sensor feature in Figure 5A. On days 0 to +6, the influenza-positive cohort lost significantly more steps than the symptoms-only cohort (Figure 5A; Table S5 in Multimedia Appendix 1). The difference in the overall ILI burden on total daily steps between the influenza-positive and symptoms-only cohorts was significant (Δ −12,764, 95% CI −16,235 to −9292; *P*<.001; Figure 5B). The influenza-positive cohort also lost significantly more active time on days 0 to +3 and +5 and experienced a significantly greater ILI burden on sleep duration on days 0 to +2 and +8 than the symptoms-only cohort (Figure 5A). Across the course of the ILI event, the change in sleep duration from baseline was 239 minutes more for the influenza-positive cohort than for the symptoms-only cohort (Δ 239, 95% CI 175.7-301.6; *P*<.001; Figure 5B). The influenza-positive cohort experienced significantly greater changes from baseline RHR on days 0 to +4 and days +7 to +9 than the symptoms-only cohort (Figure 5A). On days +1 and +2, the RHR of the influenza-positive cohort was 1.6 bpm higher than that of the symptoms-only cohort (Figure 5A).

Participants who tested positive for influenza also exhibited a longer TTRB for wearable sensor features compared with the ILI symptoms–only cohort; the TTRB was 3 days longer for total daily steps (*U*=224,057.5; *P*<.001), 1 day longer for sleep duration (*U*=224,057.5; *P*<.001), and 3 days longer for RHR δ (*U*=256,776; *P*<.001; Figure 5B).

## Discussion

### Principal Findings and Comparison With Prior Work

Our results provide evidence of the potential for wearable sensors to discriminate between individuals with and without influenza. We characterized the wearable sensor “phenotype” of participants with laboratory-confirmed influenza. Across all wearable sensor measures, the participants who tested positive for influenza exhibited greater deviations from baseline values and longer TTRB than those who reported ILI symptoms only. Changes in wearable sensor data generally emerged from the day before symptom onset and peaked on the day of or day after symptom onset. Similar observations were made using commercial wearable sensors for the detection of COVID-19, suggesting that wearable sensors may provide an objective early warning signal for the onset of ILI before individuals become aware of their symptoms [14,19,20]. Influenza is associated with greater clinical severity than other common causes of viral upper respiratory tract infections (excluding SARS-CoV-2) [30,31]. This difference in the individual experience of influenza compared with other ILIs appears to be detectable with wearable sensors. In this study, the baseline characteristics of the symptoms-only cohort did not differ meaningfully from those of the influenza-positive cohort. However, the symptoms-only cohort should not necessarily be interpreted as influenza negative because some participants with influenza infection not identified by diagnostic testing may be included in this cohort, potentially diluting the observed differences. The seasonal incidence of ILI is often estimated as 5% to 20%, although a recent study suggests that the true incidence is lower [32]. The ILI rates observed in both FluStudy2020 (1738/10,004, 17.4% of participants) and HTRI (1130/5221, 21.6% of participants) were broadly consistent with seasonal estimates.

A retrospective study of 200,000 Fitbit users suggested that wearable sensor data can predict ILI at individual and population levels [33]. Modeled ILI rates closely correlated with the Centers for Disease Control and Prevention–reported ILI rates when Fitbit data were incorporated, specifically elevated RHR (0.5 SD above user average) combined with reduced sleep efficiency (>0.5 SD below user average). Similarly, a study quantifying the population burden of ILI based on changes in activity showed that wearable sensors can be used to estimate the burden of ILI at individual and national levels in terms of the total daily step count [12]. Other studies have suggested that wearable sensors can potentially capture subtle within-person changes that could signal SARS-CoV-2 infection [14,15,18,19,21,23]. A study demonstrated differences in RHR elevations in individuals before and during COVID-19 illness compared with those with non–COVID-19 ILI [20], although another study cautioned about the potential impact of changes in wearer behavior after receiving a positive COVID-19 test result [18]. In addition, a small cohort influenza challenge study showed that when coupled with machine learning methodology, wearable sensor data can be used to predict infection status and severity [34]. Machine learning approaches coupled with wearable sensor and syndromic data have also been explored for the prediction of COVID-19 [16,21,22,35]. Our results confirm these reported changes in wearable sensor measures during an ILI event, detailing their correlation with symptom severity and duration using a large, real-world population. Moreover, our findings further underscore the potential of commercial wearable sensors to serve as a tool for the passive monitoring of well-being and infection surveillance, not just at the population level but also at the individual level.

Visual inspection of the group-level time series (Figure 2) showed ostensibly slightly more variation in day-to-day wearable sensor measures during the baseline period than during the ILI event. This may be explained by 2 factors. First, although participants’ daily lives may vary substantially during periods of health, there is a more typical trajectory of events during illness. This becomes visually apparent when participants’ data streams are aligned to their ILI onsets. Second, because the data density criteria were optimized for dense data coverage during the ILI event (ie, days −4 to +9), this period contains the greatest number of participants at each point in the time series. The larger sample size during this period results in a smaller SE of the mean (smaller error bands in Figure 2) and, in many cases, a smoother time series. In addition, there was an intriguing pattern of changes in RHR during the ILI event (Figure 2). Specifically, RHR increased just before symptom onset and remained elevated throughout the first several days of the illness before dipping below baseline levels, returning to elevated levels, and finally leveling off. Similar patterns in HR data have been reported in previous studies on influenza and COVID-19 [15,20,33,36]. At present, we do not have an evidenced clinical explanation for this phenomenon, but its persistence across studies and ILI populations suggests that it has a physiological basis rather than merely an artifactual basis.

We have previously reported that individuals (from the same data set, FluStudy2020) who sought care for their ILI experienced more severe symptoms than those who did not [37]. Here, using wearable sensor data, we show that the burden and duration of ILI were greater in participants who sought care than in those who did not. This may reflect the wearable sensor’s ability to detect a more severe disease phenotype (as with the participants who tested positive for influenza versus those who reported ILI symptoms only) rather than an independent relationship between wearable sensors and care-seeking behavior. Nonetheless, these findings suggest that wearable sensors may serve as a useful passive and noninvasive proxy for ILI severity and could, in turn, have utility in predicting care-seeking behaviors for ILI, which may help inform health care resource planning and allocation.

We also found a consistent, statistically significant though weak correlation between patient-reported severity and duration of key ILI symptoms and data from commercial wearable sensors, including the magnitude of change from baseline, cumulative burden, and TTRB of multiple wearable sensor measures. This study was conducted in a fully remote setting, using data from commercially available wearable sensors and participants’ self-reported symptoms; these data are inherently more variable than clinical measures, such as implanted medical devices, physician- or expert-reported assessments, and medical records. Although the observed correlations are not “strong” by conventional standards, they are statistically significant, coherent, and directionally meaningful.

### Limitations

This study has some limitations. First, the sample is not representative of the general population. The participants were predominantly White, female, and aged <50 years. The study design may also have had some biases owing to the inclusion criteria. For example, the participants were required to own a Fitbit device; those who own a Fitbit device may be more health conscious and physically active than the general population. Indeed, the population examined in this study had a mean daily step count (approximately 8000) that was somewhat higher than the estimates for the US adult population (approximately 6500) [33]. Although this may represent a true difference, measurement errors from the use of different sensors in different studies (eg, pedometers read fewer steps than accelerometers [33]) may provide an alternative explanation. Furthermore, the participants who lacked sufficient wearable sensor data on ILI days were excluded from the analysis; thus, the data set only included participants with strong adherence to their wearable sensor. As an exploratory observational study, we did not explicitly select a representative population; future studies may seek to improve representation. Interpersonal variation in the time of day at which the participants responded to surveys and how they interpreted the questions may have influenced the data set. As the participants reported their symptoms daily for a portion of this study, hypervigilance of their current well-being may have influenced their physical activity patterns and care-seeking behaviors (eg, observer bias).

It is noteworthy that a portion of the study period coincided with the COVID-19 pandemic, which may have also influenced care-seeking behaviors. The participants may have been more likely to delay or avoid seeking treatment owing to the overwhelmed health care services and to minimize their risk of COVID-19 infection. Moreover, COVID-19 mitigation measures may have reduced circulating influenza during the portion of the study period coinciding with the pandemic. In addition, logistical challenges were posed by the remote collection of influenza diagnostic tests, which may have resulted in miscataloging influenza-positive cases as “symptoms-only” cases. Logistical complications resulted in some influenza diagnostic kits not being delivered to the participants in a timely manner, and some testing materials became unavailable during the COVID-19 pandemic. Participants may have erroneously tested negative because of delays or errors in collecting and returning their samples to the laboratory. The timing discrepancy of influenza test kit delivery between the 2 studies also suggests that the FluStudy2020 participants may have collected their influenza test samples slightly later than the HTRI study participants, whose diagnostic kits were kept on hand rather than shipped on demand. It is not possible to estimate the impact of these factors, but their net effect is likely to be an underestimation of the magnitude of the observed differences in wearable sensor data features between participants who tested positive for influenza and those who reported symptoms only, as the population that reported symptoms only may have included individuals who were influenza positive but could not be diagnosed. Finally, nonlinear models or other machine learning techniques may be better suited for these investigations.

### Conclusions

In conclusion, using wearable sensor data, we characterized 2 cohorts of patients with ILI based on their diagnostic status and care-seeking behavior. We show distinct wearable “phenotypes” for patients who were influenza positive compared with those who reported ILI symptoms only and for care seekers compared with non–care seekers. Although our findings alone cannot be used in lieu of formal laboratory diagnostics, our results provide further supportive evidence of the potential for commercial wearable sensors to serve as an early detection system for influenza infections, whereby device users may receive a digital suggestion to seek care, to undergo diagnostic testing, or to even quarantine. Wearable sensors also have the potential to discriminate care-seeking from non–care-seeking populations, which could help inform health care resource planning in the future. Future studies validating early detection and care-seeking prediction algorithms are needed.

## Appendix

Multimedia Appendix 1 Supplementary tables showing: the daily influenza-like illness (ILI) burden for the overall study population (Table S1); baseline characteristics (Table S2) and the difference in daily ILI burden (Table S3) for participants who attended a healthcare visit compared with those who did not; baseline characteristics (Table S4) and the difference in daily ILI burden (Table S5) for participants with confirmed influenza infection compared with those who had ILI symptoms only. The overall ILI burden for total steps and sleep duration for the wearable analysis population is shown in Figure S1.

## Abbreviations

bpm: beats per minute
HR: heart rate
HTRI: Home Testing of Respiratory Illness
ILI: influenza-like illness
PRO: patient-reported outcome
QoL: quality of life
RHR: resting heart rate
SIP: shelter in place
TTRB: time to return to baseline

## Data Availability

The data from the Home Testing of Respiratory Illness study are available on the internet [38]. Requests for access to the data sets and custom code generated, used, or analyzed in this study should be directed to the corresponding author.
