# Supplementary material for: Characterization of Influenza-Like Illness Burden Using Commercial Wearable Sensor Data and Patient-Reported Outcomes: Mixed Methods Cohort Study
Source: J Med Internet Res. 2023 Mar 23;25:e41050. doi: 10.2196/41050 (PMC10131710; doi:10.2196/41050)
Supplement: Multimedia Appendix 1 [file jmir_v25i1e41050_app1.pdf]

Multimedia appendix.

Table S1 Daily ILI burden for the overall study population

ILI day	Change from baseline	95% CI	*P* value	q-value
		**Total steps**		
**-4**	123	-65 to 311	0.2	>0.9
**-3**	72	-116 to 259	0.5	>0.9
**-2**	-213	-400 to -27	0.025	0.3
**-1**	-1,053	-1,238 to -868	<0.001	<0.001
**0**	-2,515	-2,699 to -2,331	<0.001	<0.001
**1**	-2,139	-2,324 to -1,953	<0.001	<0.001
**2**	-1,665	-1,851 to -1,479	<0.001	<0.001
**3**	-1,277	-1,463 to -1,092	<0.001	<0.001
**4**	-1,017	-1,203 to -831	<0.001	<0.001
**5**	-744	-930 to -559	<0.001	<0.001
**6**	-656	-842 to -470	<0.001	<0.001
**7**	-478	-664 to -293	<0.001	<0.001
**8**	-400	-586 to -214	<0.001	<0.001
**9**	-326	-512 to -140	<0.001	0.008
		**Proportion of day spent active with >50 spm**		
**-4**	0	0.00–0.01	0.3	>0.9
**-3**	0	0.00–0.00	0.8	>0.9
**-2**	0	-0.01 to 0.00	0.2	>0.9
**-1**	-0.01	-0.02 to -0.01	<0.001	<0.001
**0**	-0.04	-0.04 to -0.04	<0.001	<0.001

**1**	-0.04	-0.04 to -0.03	<0.001	<0.001
**2**	-0.03	-0.04 to -0.03	<0.001	<0.001
**3**	-0.02	-0.03 to -0.02	<0.001	<0.001
**4**	-0.02	-0.02 to -0.02	<0.001	<0.001
**5**	-0.01	-0.02 to -0.01	<0.001	<0.001
**6**	-0.01	-0.01 to -0.01	<0.001	<0.001
**7**	-0.01	-0.01 to 0.00	0.015	0.2
**8**	0	-0.01 to 0.00	0.12	>0.9
**9**	0	-0.01 to 0.00	0.2	>0.9
		**Sleep duration**		
**-4**	-1	-7.1 to 5.1	0.7	>0.9
**-3**	-0.85	-6.9 to 5.2	0.8	>0.9
**-2**	1.8	-4.3 to 7.8	0.6	>0.9
**-1**	10	4.4–16	<0.001	0.01
**0**	43	37–49	<0.001	<0.001
**1**	43	37–49	<0.001	<0.001
**2**	17	11–23	<0.001	<0.001
**3**	11	5.4–17	<0.001	0.003
**4**	9.2	3.2–15	0.003	0.036
**5**	11	5.4–17	<0.001	0.003
**6**	8.9	2.9–15	0.004	0.053
**7**	6.8	0.76–13	0.027	0.4
**8**	8.1	2.1–14	0.008	0.12
**9**	11.0	5.0–17	<0.001	0.004
		**Sleep efficiency**		
**-4**	-0.09	-0.35 to 0.18	0.5	>0.9

**-3**	-0.14	-0.40 to 0.12	0.3	>0.9
**-2**	-0.35	-0.61 to -0.09	0.009	0.13
**-1**	-0.72	-1.0 to -0.46	<0.001	<0.001
**0**	-2.3	-2.6 to -2.1	<0.001	<0.001
**1**	-1.9	-2.2 to -1.6	<0.001	<0.001
**2**	-1.1	-1.4 to -0.85	<0.001	<0.001
**3**	-0.8	-1.1 to -0.54	<0.001	<0.001
**4**	-0.58	-0.84 to -0.32	<0.001	<0.001
**5**	-0.38	-0.64 to -0.12	0.004	0.059
**6**	-0.09	-0.36 to 0.17	0.5	>0.9
**7**	-0.21	-0.47 to 0.05	0.12	>0.9
**8**	0.07	-0.19 to 0.33	0.6	>0.9
**9**	0.08	-0.18 to 0.34	0.6	>0.9
		**RHR**		
**-4**	0.04	-0.10 to 0.19	0.6	>0.9
**-3**	0.13	-0.02 to 0.27	0.085	>0.9
**-2**	0.32	0.18–0.47	<0.001	<0.001
**-1**	0.73	0.59–0.87	<0.001	<0.001
**0**	1.4	1.3–1.6	<0.001	<0.001
**1**	1.8	1.6–1.9	<0.001	<0.001
**2**	1.7	1.5–1.8	<0.001	<0.001
**3**	1.4	1.2–1.5	<0.001	<0.001
**4**	1	0.89–1.2	<0.001	<0.001
**5**	0.61	0.47–0.75	<0.001	<0.001
**6**	0.29	0.15–0.43	<0.001	0.001
**7**	-0.02	-0.16 to 0.12	0.80	>0.9

**8**	-0.23	-0.37 to -0.09	0.002	0.02
**9**	-0.31	-0.45 to -0.17	<0.001	<0.001

CI, confidence interval; ILI, influenza-like illness; RHR, resting heart rate; spm, steps per minute.

Table S2 Cohort comparison of participants who attended a healthcare visit vs. those who did not

***n* (%)**	**Overall *N* = 1,998**	**Healthcare visit *N* = 618**	**No healthcare visit *N* = 1,380**	***P* value**	**q-value**
Age	—	—	—	0.008	0.017
18–49 years	1,787 (89.4)	533 (86.2)	1,254 (90.9)	—	—
50–64 years	197 (9.9)	80 (12.9)	117 (8.5)	—	—
65+ years	14 (0.7)	5 (0.8)	9 (0.7)	—	—
Sex	—	—	—	0.2	0.3
Male	234 (11.7)	73 (11.8)	161 (11.7)	—	—
Female	1,751 (87.6)	544 (88.0)	1,207 (87.5)	—	—
Non-binary	13 (0.7)	1 (0.2)	12 (0.9)	—	—
Race	—	—	—	0.12	0.2
American Indian or Alaska Native	4 (0.2)	2 (0.3)	2 (0.1)	—	—
Asian	44 (2.2)	9 (1.5)	35 (2.5)	—	—
Black or African American	60 (3.0)	27 (4.4)	33 (2.4)	—	—

Native Hawaiian or other Pacific Islander	3 (0.2)	1 (0.2)	2 (0.1)	–	–
White	1,795 (89.8)	552 (89.3)	1,243 (90.1)	–	–
Multiple races	73 (3.7)	20 (3.2)	53 (3.8)	–	–
Other	19 (1.0)	7 (1.1)	12 (0.9)	–	–
American Region	–	–	–	0.063	0.1
Northeast	359 (18.0)	114 (18.4)	245 (17.8)	–	–
South	603 (30.2)	205 (33.2)	398 (28.8)	–	–
Midwest	664 (33.2)	202 (32.7)	462 (33.5)	–	–
West	371 (18.6)	96 (15.5)	275 (19.9)	–	–
Unknown	1 (0.1)	1 (0.2)	0 (0)	–	–
BMI[a]	–	–	–	0.3	0.4
Normal	466 (23.3)	129 (20.9)	337 (24.4)	–	–
Obese	967 (48.4)	313 (50.6)	654 (47.4)	–	–
Overweight	544 (27.2)	171 (27.7)	373 (27.0)	–	–
Underweight	19 (1.0)	5 (0.8)	14 (1.0)	–	–
Unknown	2 (0.1)	0 (0)	2 (0.1)	–	–
Criteria	–	–	–	–	–
Influenza positive	357 (17.9)	160 (25.9)	197 (14.3)	<0.001	<0.001

Diagnosed	259 (13.0)	258 (41.7)	1 (<0.1)	<0.001	<0.001
Prescribed antiviral	208 (10.4)	192 (31.1)	16 (1.2)	<0.001	<0.001
Baloxavir marboxil	26 (1.3)	24 (3.9)	2 (0.1)	<0.001	<0.001
Oseltamivir phosphate	186 (9.3)	172 (27.8)	14 (1.0)	<0.001	<0.001
Data validity	–	–	–	–	–
Valid steps data	1,909 (95.5)	589 (95.3)	1,320 (95.7)	0.8	0.9
Valid sleep data	1,908 (95.5)	591 (95.6)	1,317 (95.4)	>0.9	>0.9
Valid heart rate data	1,787 (89.4)	566 (91.6)	1,221 (88.5)	0.044	0.082

[a]BMI categories were defined as follows: underweight, BMI < 18.5; normal, BMI = 18.5–24.9; overweight, BMI = 25.0–29.9; obese, BMI ≥ 30.0. BMI, body mass index.

Table S3 The difference in day-by-day ILI burden between participants who did and did not seek healthcare

ILI day	Difference between non-healthcare-seeking and healthcare-seeking participants	95% CI	*P* value	q-value
**Total steps**				
**-4**	30	-413 to 473	0.9	>0.9
**-3**	-60	-503 to 382	0.8	>0.9
**-2**	-12	-452 to 428	>0.9	>0.9
**-1**	-512	-950 to -74	0.022	0.3
**0**	-1,077	-1,513 to -641	<0.001	<0.001
**1**	-1,765	-2,205 to -1,324	<0.001	<0.001
**2**	-1,481	-1,922 to -1,040	<0.001	<0.001
**3**	-1,338	-1,779 to -898	<0.001	<0.001
**4**	-1,312	-1,752 to -871	<0.001	<0.001
**5**	-856	-1,297 to -415	<0.001	0.002
**6**	-539	-980 to -98	0.017	0.2
**7**	-610	-1,050 to -171	0.007	0.092
**8**	-774	-1,213 to -334	<0.001	0.008
**9**	-771	-1,211 to -331	<0.001	0.008
**Proportion of day active with > 50 spm**				
**-4**	0	-0.01 to 0.01	0.6	>0.9
**-3**	-0.01	-0.02 to 0.01	0.3	>0.9
**-2**	0	-0.01 to 0.01	0.9	>0.9

**-1**	-0.01	-0.02 to 0.00	0.027	0.4
**0**	-0.02	-0.03 to -0.01	<0.001	0.01
**1**	-0.03	-0.04 to -0.02	<0.001	<0.001
**2**	-0.02	-0.03 to -0.01	<0.001	<0.001
**3**	-0.02	-0.03 to -0.01	<0.001	<0.001
**4**	-0.02	-0.03 to -0.01	<0.001	<0.001
**5**	-0.02	-0.03 to -0.01	0.002	0.028
**6**	-0.01	-0.02 to 0.00	0.005	0.07
**7**	-0.01	-0.02 to 0.00	0.008	0.11
**8**	-0.02	-0.03 to -0.01	0.001	0.014
**9**	-0.02	-0.03 to -0.01	0.003	0.036
**Sleep duration**				
**-4**	-3.5	-18 to 11	0.6	>0.9
**-3**	2.3	-12 to 17	0.8	>0.9
**-2**	4.6	-9.7 to 19	0.5	>0.9
**-1**	7.2	-7.1 to 21	0.3	>0.9
**0**	15	1.1–29	0.035	0.5
**1**	22	8.0–36	0.002	0.032
**2**	26	12–40	<0.001	0.005
**3**	2.1	-12 to 16	0.8	>0.9
**4**	20	5.7–34	0.006	0.085
**5**	16	1.3–30	0.032	0.4
**6**	3.5	-11 to 18	0.6	>0.9
**7**	11	-2.9 to 26	0.12	>0.9
**8**	13	-1.6 to 27	0.081	>0.9
**9**	21	7.1–36	0.003	0.048

**Sleep efficiency**				
**-4**	0.26	-0.37 to 0.88	0.4	>0.9
**-3**	0.32	-0.30 to 0.95	0.3	>0.9
**-2**	0.11	-0.51 to 0.73	0.7	>0.9
**-1**	0.42	-0.20 to 1.0	0.2	>0.9
**0**	-1	-1.7 to -0.44	<0.001	0.011
**1**	-1.1	-1.7 to -0.46	<0.001	0.009
**2**	-0.29	-0.91 to 0.33	0.4	>0.9
**3**	-0.36	-1.0 to 0.26	0.3	>0.9
**4**	-0.2	-0.82 to 0.42	0.5	>0.9
**5**	-0.19	-0.81 to 0.44	0.6	>0.9
**6**	0.15	-0.47 to 0.77	0.6	>0.9
**7**	0.09	-0.53 to 0.71	0.8	>0.9
**8**	0.29	-0.33 to 0.90	0.4	>0.9
**9**	0.4	-0.22 to 1.0	0.2	>0.9
**RHR**				
**-4**	-0.12	-0.47 to 0.22	0.5	>0.9
**-3**	-0.13	-0.47 to 0.21	0.5	>0.9
**-2**	-0.14	-0.49 to 0.20	0.4	>0.9
**-1**	0	-0.34 to 0.34	>0.9	>0.9
**0**	0.35	0.01–0.69	0.041	0.6
**1**	0.65	0.31–1.0	<0.001	0.002
**2**	0.54	0.20–0.88	0.002	0.026
**3**	0.41	0.07–0.75	0.018	0.3
**4**	0.23	-0.11 to 0.57	0.2	>0.9
**5**	0.09	-0.25 to 0.43	0.6	>0.9

**6**	-0.17	-0.51 to 0.17	0.3	>0.9
**7**	-0.36	-0.70 to -0.02	0.038	0.5
**8**	-0.49	-0.83 to -0.15	0.004	0.062
**9**	-0.4	-0.74 to -0.06	0.023	0.3

CI, confidence interval; ILI, influenza-like illness; RHR, resting heart rate; spm, steps per minute.

Table S4 Cohort comparison of participants with confirmed influenza infection vs. those with ILI symptoms only

***n* (%)**	**Overall *N* = 2,388**	**Influenza positive *N* = 364**	**Symptoms criteria only *N* = 2,024**	***P* value**	**q-value**
Age	–	–	–	0.3	0.3
18–49 years	2,143 (89.7)	318 (87.4)	1,825 (90.2)	–	–
50–64 years	229 (9.6)	43 (11.8)	186 (9.2)	–	–
65+ years	16 (0.7)	3 (0.8)	13 (0.6)	–	–
Sex	–	–	–	0.003	0.006
Male	336 (14.1)	71 (19.5)	265 (13.1)	–	–
Female	2,039 (85.4)	293 (80.5)	1,746 (86.3)	–	–
Non-binary	13 (0.5)	0 (0)	13 (0.6)	–	–
Race	–	–	–	0.7	0.7
American Indian or Alaska Native	7 (0.3)	0 (0)	7 (0.3)	–	–
Asian	64 (2.7)	11 (3.0)	53 (2.6)	–	–
Black or African American	75 (3.1)	9 (2.5)	66 (3.3)	–	–

Native Hawaiian or other Pacific Islander	3 (0.1)	0 (0)	3 (0.1)	–	–
White	2,132 (89.3)	332 (91.2)	1,800 (88.9)	–	–
Multiple races	83 (3.5)	8 (2.2)	75 (3.7)	–	–
Other	24 (1.0)	4 (1.1)	20 (1.0)	–	–
American Region	–	–	–	<0.001	0.002
Northeast	437 (18.3)	59 (16.2)	378 (18.7)	–	–
South	705 (29.5)	106 (29.1)	599 (29.6)	–	–
Midwest	786 (32.9)	150 (41.2)	636 (31.4)	–	–
West	459 (19.2)	49 (13.5)	410 (20.3)	–	–
Unknown	1 (<0.1)	0 (0)	1 (<0.1)	–	–
BMI[a]	–	–	–	0.3	0.4
Normal	541 (22.7)	90 (24.7)	451 (22.3)	–	–
Obese	1,155 (48.4)	161 (44.2)	994 (49.1)	–	–
Overweight	669 (28.0)	111 (30.5)	558 (27.6)	–	–
Underweight	21 (0.9)	2 (0.5)	19 (0.9)	–	–
Unknown	2 (0.1)	0 (0)	2 (0.1)	–	–
Criteria	–	–	–	–	–
Diagnosed	260 (10.9)	110 (30.2)	150 (7.4)	<0.001	<0.001

Prescribed antiviral	238 (10.0)	91 (25.0)	147 (7.3)	<0.001	<0.001
Baloxavir marboxil	28 (1.2)	8 (2.2)	20 (1.0)	0.061	0.12
Oseltamivir phosphate	214 (9.0)	84 (23.1)	130 (6.4)	<0.001	<0.001
Data validity	–	–	–	–	–
Valid steps data	2,282 (95.6)	341 (93.7)	1,941 (95.9)	0.08	0.14
Valid sleep data	2,276 (95.3)	353 (97.0)	1,923 (95.0)	0.13	0.2
Valid heart rate data	2,165 (90.7)	334 (91.8)	1,831 (90.5)	0.5	0.5

[a]BMI categories were defined as follows: underweight, BMI < 18.5; normal, BMI = 18.5–24.9; overweight, BMI = 25.0–29.9; obese, BMI ≥ 30.0. BMI, body mass index; ILI, influenza-like illness.

Table S5 The difference in day-by-day ILI burden between participants who had ILI symptoms only and those who tested positive for influenza

ILI day	Difference between ILI symptoms only and influenza+ cohorts	95% CI	*P* value	q-value
**Total steps**				
**-4**	22	-503 to 548	>0.9	>0.9
**-3**	-40	-565 to 485	0.9	>0.9
**-2**	54	-469 to 577	0.8	>0.9
**-1**	-508	-1,029 to 13	0.056	0.8
**0**	-2,000	-2,519 to -1,481	<0.001	<0.001
**1**	-2,384	-2,907 to -1,861	<0.001	<0.001
**2**	-1,685	-2,208 to -1,161	<0.001	<0.001
**3**	-1,498	-2,021 to -975	<0.001	<0.001
**4**	-1,261	-1,786 to -737	<0.001	<0.001
**5**	-1,002	-1,525 to -480	<0.001	0.002
**6**	-781	-1,304 to -257	0.003	0.048
**7**	-732	-1,255 to -210	0.006	0.084
**8**	-636	-1,159 to -113	0.017	0.2
**9**	-754	-1,278 to -229	0.005	0.068
**Proportion of day active with > 50 spm**				
**-4**	0.01	-0.01 to 0.02	0.4	>0.9
**-3**	0	-0.01 to 0.01	>0.9	>0.9
**-2**	0	-0.01 to 0.01	0.6	>0.9
**-1**	0	-0.01 to 0.01	0.7	>0.9
**0**	-0.03	-0.05 to -0.02	<0.001	<0.001

**1**	-0.05	-0.06 to -0.04	<0.001	<0.001
**2**	-0.03	-0.04 to -0.02	<0.001	<0.001
**3**	-0.03	-0.04 to -0.02	<0.001	<0.001
**4**	-0.02	-0.03 to -0.01	0.005	0.067
**5**	-0.02	-0.03 to -0.01	<0.001	0.009
**6**	-0.01	-0.02 to 0.00	0.083	>0.9
**7**	-0.01	-0.02 to 0.00	0.2	>0.9
**8**	-0.01	-0.02 to 0.00	0.1	>0.9
**9**	-0.01	-0.02 to 0.00	0.15	>0.9
**Sleep duration**				
**-4**	-0.42	-17 to 16	>0.9	>0.9
**-3**	1.2	-16 to 18	0.9	>0.9
**-2**	6	-11 to 23	0.5	>0.9
**-1**	0.26	-16 to 17	>0.9	>0.9
**0**	26	9.8–43	0.002	0.025
**1**	52	35–68	<0.001	<0.001
**2**	33	17–50	<0.001	0.001
**3**	12	-4.5 to 29	0.2	>0.9
**4**	19	2.7–36	0.023	0.3
**5**	14	-2.3 to 31	0.092	>0.9
**6**	16	-0.67 to 33	0.06	0.8
**7**	14	-3.1 to 30	0.11	>0.9
**8**	25	8.8–42	0.003	0.038
**9**	20	2.9–36	0.022	0.3
**Sleep efficiency**				
**-4**	-0.18	-0.90 to 0.55	0.6	>0.9

**-3**	-0.26	-1.0 to 0.47	0.5	>0.9
**-2**	-0.35	-1.1 to 0.38	0.3	>0.9
**-1**	-0.51	-1.2 to 0.21	0.2	>0.9
**0**	-1.5	-2.2 to -0.81	<0.001	<0.001
**1**	-1.8	-2.5 to -1.1	<0.001	<0.001
**2**	-1.2	-1.9 to -0.46	0.001	0.019
**3**	-0.82	-1.5 to -0.10	0.026	0.4
**4**	-0.78	-1.5 to -0.05	0.036	0.5
**5**	-0.28	-1.0 to 0.44	0.4	>0.9
**6**	0.27	-0.45 to 1.0	0.5	>0.9
**7**	-0.05	-0.78 to 0.68	0.9	>0.9
**8**	-0.46	-1.2 to 0.26	0.2	>0.9
**9**	-0.22	-0.95 to 0.50	0.6	>0.9
		**RHR**		
**-4**	-0.31	-0.70 to 0.09	0.13	>0.9
**-3**	-0.33	-0.72 to 0.07	0.11	>0.9
**-2**	-0.31	-0.70 to 0.09	0.13	>0.9
**-1**	0.29	-0.10 to 0.68	0.2	>0.9
**0**	1	0.79–1.6	<0.001	<0.001
**1**	2	1.3–2.0	<0.001	<0.001
**2**	2	1.2–2.0	<0.001	<0.001
**3**	1	0.76–1.5	<0.001	<0.001
**4**	1	0.25–1.0	0.001	0.02
**5**	0	-0.35 to 0.43	0.8	>0.9
**6**	-0.53	-0.92 to -0.14	0.008	0.12
**7**	-1	-1.4 to -0.59	<0.001	<0.001

**8**	-1.2	-1.5 to -0.76	<0.001	<0.001
**9**	-1.2	-1.6 to -0.84	<0.001	<0.001

CI, confidence interval; ILI, influenza-like illness; RHR, resting heart rate;

spm, steps per minute.

Figure S1 Overall ILI burden measured by wearable device for (a) total steps, and (b) sleep duration for the wearable analysis population.

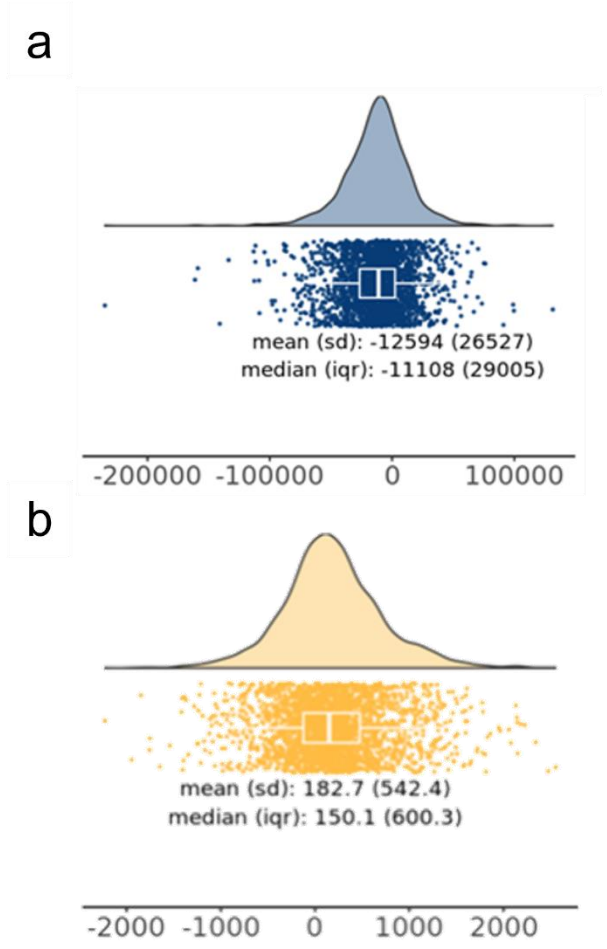

iqr, interquartile range; sd, standard deviation.
